# The jaguar and the PhD

**DOI:** 10.1371/journal.pbio.2004152

**Published:** 2018-02-07

**Authors:** Sergio Avila-Villegas

**Affiliations:** Sierra Club, Tucson, Arizona, United States of America

This Perspective is part of the *Conservation Stories from the Front Lines Collection*

From a young age, I dreamed of studying big cats. My favorite cat was the African lion, and I had a recurring dream of having one in my backyard, which I fed bones. This love of cats (and wildlife in general) started with books, encyclopaedias, nature shows, and zoos. I eventually earned a Master’s in Science degree with a study on mountain lions in Baja California, Mexico. I produced a map with preferred habitat; analyzed scat contents to identify mountain lions’ prey species; and conducted numerous surveys of the local residents about economic activities, cattle grazing, and ranching activities. Not surprisingly, the results pointed to conflict in areas where humans or their activities converge with mountain lion distribution.

Some years later, I was invited to be part of a new study of jaguars in Northwest Mexico. The study aimed to generate new information about the social organization and feeding habits of jaguars and mountain lions in the Sierra Madre Mountains of Sonora and about their dispersal movements into ranges along the United States–Mexico border, where they had been recently documented. The study would be the first of its type for this region, gathering new data with radio telemetry, remote camera photographs, scat, tracks, and sign data points collected in the field. The study area is about 120 miles south of the Arizona–Sonora border—a remote, rugged area of the Sierra Madre with low human population density and historically a region of extensive private lands, mostly dedicated to cattle grazing by private landowners. This project would be my PhD dissertation.

Tropical and temperate species come together, at least for a season, in this transition area between the Sonoran Desert scrub and the oak and pine woodlands of higher elevation ranges on the western slope of the Sierra Madre, where canyons and streams lined with palm trees grow next to columnar cacti and wildflowers. The area sustains a great diversity of plants and animals at the edge of the Nearctic and Neotropical regions—bald eagles returning to places where military macaws live; ocelots moving through the same drainages as coatis and desert tortoises.

The “research station” was located at the end of a long, winding dirt road, about 50 miles (80 km) from the closest town. It took at least nine hours to drive there. The remoteness meant living in very basic conditions and having simple arrangements for activities like sleeping, showering, and cooking—not the typical National Geographic or Discovery Channel adventures. Before my arrival, remote cameras were installed along creeks, game trails, and places where jaguar tracks or kills were found. Additionally, foot-hold snares—long metal cables attached to the base of a tree, with a loop and swivel at the other end where a jaguar or a mountain lion would walk in and get caught—were also set in order to capture them to place radio collars and monitor the big cats’ movements.

I quickly realized the prospect of a meaningful research study and, at the same time, the challenge it presented. I was ready to commit to this project over the long term, gather and analyze new data, write a superlative thesis, and publish and disseminate the findings of the project to help inform jaguar conservation. I was about to help jaguar conservation, as I always dreamed I could do. Daily life was a routine of driving and hiking to get telemetry locations of two already-collared mountain lions and check remote cameras and snares along several trails. The simplicity of life meant that any project analyses (for example, plotting the telemetry locations on a map) were done using basic tools like a compass, a topographic map, and a ruler. Creating this map of daily movements of two mountain lions for a few weeks allowed the crew to visualize the lions’ territory and preferred movement corridors and to find their cached prey. Every night after fieldwork, the team would discuss what we found, interpret it, and put it together based on jaguar and mountain lion natural history. I located dots on a topographic map representing collar locations, tracks, kills, and scat. I studied the contour lines and found ways to connect the dots for the cats’ movements. Our field observations were creating a picture of big cat territories and movement, and our knowledge of the area expanded quickly—a jaguar track here, a cached mountain lion kill there, scat containing javelina hair over there. This was an exciting opportunity for a ground-breaking field study culminating in my PhD degree. It was a dream come true to study a species that was little known in the region and understand their requirements in an area where they have never been studied—the US–Mexico borderlands. The implications of this study would be relevant for binational jaguar conservation, given recent jaguar sightings in Arizona and New Mexico, and a renewed effort to recover jaguars in their historic ranges by conservationists from both countries.

After two months of familiarizing ourselves with the area, we started expanding the snares and remote camera networks, and we worked in teams to cover more ground. The field crew was ambitious and committed—we hiked long distances to survey new areas, collect track and sign data, and locate new remote cameras. We captured and collared two more mountain lions, and our daily telemetry readings multiplied.

I was checking traps by myself when I discovered the first jaguar captured, a female, on a warm spring morning. I was familiar with the trail, the trees, even my own tracks from the day before. As I kept walking, coming to a bend in the canyon, something was out of order and didn’t feel right. I kept walking slowly and saw a bush moving; a big cloud of dust arose, and a deep, guttural sound came from behind the bush. It was the roar of a jaguar.

She was looking directly at me, ears back, teeth showing, only 50 feet from where I was, pulling my camera from my backpack, shaking. Seeing a jaguar for the first time in my life was a dream came true, and also a realization that my wish to study jaguars did not match their own desire to be left alone and continue their normal lives. But I knew I was doing it for the right cause and felt responsible for this cat’s safety.

She was energetic, angry. She didn’t want to be there: she was pulling on the cable, roaring, hissing, and hitting the ground, rocks, and sticks. This behavior contrasted with that of mountain lions we captured before: somehow shy, hissing but not very aggressive, and, after being anesthetized, allowing us to remove the snares, set a radio collar, and take measurements quickly. Jaguars responded quite differently. The female was anesthetized with a mix of ketamine and xylazine. We darted her and quickly released the snared leg, checked her physical condition, cooled her down, and set the radio collar under the shade. During this process, I discovered she was lactating and that there was possibly at least one cub waiting for her close by.

We left her to rest and recover safely and watched her for hours. She slowly moved in the direction of the hillside, and we left her to reunite with her cub(s). Over the following days, using the telemetry receiver, we confirmed she had safely moved from the capture site.

We celebrated the successful capture and collaring of the first female jaguar in the region, which would generate new information about the females’ territorial, reproductive, and feeding habits. The outcomes of this would be so important for jaguar conservation. This project was on the right track. However, none outside of the field crew knew we had captured a jaguar. Due to the very limited cell phone reception, we were not able to share the news with other people in the project, and we knew this was important news that needed to be communicated, so we decided to close all snares temporarily, travel back to town, and call our supervisor with the good news. Two days after the female’s capture, we loaded the car and went out on our regular hikes to close all the snares before leaving to town in order to close the snares and avoid capturing any animals when we weren’t there to release them. We were giddy, excited to share the news, so we divided the work and went on to different trails. It was during this hike that we found a second jaguar snared.

The male jaguar was captured in a snare set up less than a week before, near a horse that had recently died of natural causes. We set the snares around the horse based on the observation made with remote cameras that jaguars scavenge and consume prey they did not kill—another piece of information about jaguars coming from this study. This jaguar was bigger and more aggressive than the first. A hind leg was captured in the snare, which gave room for this jaguar to run and pull on the cable, clearly hurting himself trying to release the leg.

It was the first day of April, and the temperature quickly passed 90 degrees in the morning. The jaguar was very difficult to approach and anesthetise, while overheating due to its excitement. He seemed to tense his muscles when he heard the blowgun, and darts were bouncing off. I distracted him by coming to the side and making him face me so the dart could be shot into his rump. I was 10 feet from an adult male jaguar that wanted to escape, kill me, and never be seen again. I knew I was doing the right thing for his species, even though he was not very interested in my upcoming scientific dissertation.

Finally, the jaguar calmed down enough for us to approach him, cover his eyes, and move him to the shade under a large oak tree. We administered more drugs because the jaguar was still moving; measuring and weighing were difficult tasks to complete. The jaguar never fell asleep completely. We finished processing and left him in the shade to recover and move away. He moved a little and seemed to be doing well, so we left him to fully recover overnight. We decided to stay one more day to make sure the jaguar had recovered before leaving to town to share the news of the two jaguars captured and collared in three days. The following morning, car loaded and spirits high, we went to check on him before leaving to town.

We parked the car close to the capture site and used the telemetry receiver to find the jaguar’s signal. The signal came from a different location, close to a large cliff not too far from the site, and seemed to be the normal “beep-beep” signal of a moving animal. He was close; the signal was strong. We moved slowly to try to get a visual of the cat, assuming he was resting under the cliff. Another reading and the signal sounded stronger, but it had moved from the cliff closer to the original capture spot. We moved slowly, following the signal and looking through binoculars; then, the signal changed, and the beeps changed pace to a faster “beep-beep-beep.”

My heart sunk when we found the jaguar lying on his side motionless under the tree where we left him the day before. That “beep-beep-beep” was a mortality signal, and by now, it was strong. Our movements made enough noise for the jaguar to hear, but he didn’t react. This was the one thing we never expected to happen in this project: a big, roaring cat, the largest cat in the Americas, the cat I had always dreamed of studying and protecting, dead. He had died overnight.

It has taken me over a decade to absorb and analyze this outcome. Losing a jaguar that was full of life right in front of me was a huge blow to my career and personal life. I realized I had lost sight of the goal—jaguar conservation—and instead was following a less important goal: getting a PhD degree, writing a dissertation, and publishing an article. I justified our actions based on new science—generating new information, collecting data, and conducting analyses of their movements and feeding. And I had been so focused on the route I needed to follow to collect data, analyze it, and receive my degree that I had stopped paying attention to the animals I was trying to study.

That jaguar didn’t make it because I never considered what he wanted. His death had no justification in my mind. And seeing him dead changed my life. That jaguar was the subject of my research, and the subject of my research was now dead. How could I protect jaguars if they were going to die when I tried to study them?

The death of this jaguar was not only a heavy responsibility and loss for my own project and personal goals. It created conflict among the field crew, with our supervisor, and with the larger group of organizations and individuals peripherally involved in the project. Different people saw this death in different ways, from “Mistakes happen, no big deal” to “I can’t believe you guys let this jaguar die” to “I will never be a biologist again.” Clearly, our ultimate goals were not the same.

I now believe that jaguar died to teach us a lesson (not just those of us in the field crew, but “us” in the larger scientific and conservation community). It’s a lesson about ethics, about scientific research justifying any methods or outcomes. It’s a lesson about the costs of ignoring basic facts about our study subjects. And it’s a lesson about ignoring the fact that there might be safer, cheaper, or less stressful study methods for both the animals and the researchers.

The personal and professional impact of having a jaguar die after a capture has been profound, starting with the fact that I lost my PhD project, data, and advisor. After conflicts ensued, blame was directed at different people, and mediation meetings were needed. It took me months to process and return to my work on conservation. I decided to continue my work as a field biologist and share the painful lessons from my experience with others, including biologists, volunteers, and citizen scientists.

I started promoting non-invasive methods, like remote cameras, track and sign identification, and scat analysis to study wildlife safely. Non-invasive methods provide information from a wide variety of animals, allowing the documentation of many species (remote cameras: land-based mid- and large-sized animals; tracking: many species) and their interactions (hunting, feeding, territorial marking, family/group size, age groups). I received tracking certifications and then trained dozens of biologists, citizen scientists, volunteers. and college students to identify tracks, scrapes, and kills, especially those of jaguars, mountain lions, ocelots, bobcats, black bears, Mexican gray wolves, and coyotes, without ever having to touch one. I emphasized to them that although this type of research doesn’t result in as much information as a radio collar, it’s also not going to place them or their study subjects at risk.

Moreover, this new approach helped me see the bigger picture of how scientific research and applied conservation can address immediate issues on the ground. While working with landowners and deploying remote cameras and tracking teams across a large region, I was able to identify healthy wildlife populations, habitats, and corridors and document a wide array of species. Through these efforts, I was able to see the power of collaboration between public and private groups and individuals to document and monitor wildlife, educate the public, and learn from each participant’s experience. This collaborative work also allowed me to address local people’s needs and priorities instead of deciding on abstract project goals that only served a typical research model.

Working alongside landowners to survey new areas in search of jaguar corridors, I found a place where common and protected species live, thrive, and complete their life cycles, from jaguars and golden eagles to large deer and javelina herds and numerous butterfly species. In this site, located along a sinuous canyon where two creeks merged under oak, juniper, ash, and sycamore trees, we followed seasonal changes by remote camera: dry or rainy season, creek running, grass growing. The remote camera revealed the presence of resident, migrant, nonprotected, and protected species, including jaguars. The images from remote cameras told me that healthy habitats would mean the protection of species living there, including jaguars. The results from the surveys were so successful in one property that the owner brought the inventories to government officials and asked to protect his property. Several months later, Mexico’s National Commission of Natural Protected Areas designated this property as a wildlife reserve under a “Voluntary Land Conservation” model.

Today, I work in the Sonoran Desert of the Southwestern United States and Northwest Mexico, connecting conservation efforts and the people who work to study and protect an area encompassing thousands of square miles. Here, government and nongovernment groups, along with private landowners and academics, work to study and protect shared resources and address shared threats. My work is more that of a “catalyst” for conservation, connecting people, facilitating conversations, and empowering local communities to address specific needs while maintaining wildlife corridors and restoring healthy habitats and even addressing climate change threats.

The projects I am involved in are not “my own” projects, but rather those of a group of interdisciplinary scientists, local peoples, and conservation groups. This is not pure science but applied conservation science. Having a PhD degree is not on my radar anymore, but jaguars are. I continue to learn from my failures, sharing the pain and knowledge gained and encouraging young scientists to expand the way they think from classic scientific research to a more applied model of conservation.

